# Flowering-Related RING Protein 1 (*FRRP1*) Regulates Flowering Time and Yield Potential by Affecting Histone H2B Monoubiquitination in Rice (*Oryza Sativa*)

**DOI:** 10.1371/journal.pone.0150458

**Published:** 2016-03-02

**Authors:** Yiwei Du, Wei He, Changwang Deng, Xi Chen, Lanming Gou, Fugui Zhu, Wei Guo, Jianfu Zhang, Tao Wang

**Affiliations:** 1 State Key Laboratory of Agrobiotechnology, College of Biological Sciences, China Agricultural University, Beijing, 100193, China; 2 Rice Research Institute, Fujian Academy of Agricultural Sciences/ Key Laboratory of Hybrid Rice Germplasm Enhancement and Molecular Breeding in South China, Ministry of Agriculture, Fuzhou, China; Institute of Genetics and Developmental Biology, Chinese Academy of Sciences, CHINA

## Abstract

Flowering time is a critical trait for crops cultivated under various temperature/photoperiod conditions around the world. To understand better the flowering time of rice, we used the vector pTCK303 to produce several lines of RNAi knockdown transgenic rice and investigated their flowering times and other agronomic traits. Among them, the heading date of *FRRP1*-RNAi knockdown transgenic rice was 23–26 days earlier than that of wild-type plants. *FRRP1* is a novel rice gene that encodes a C3HC4-type Really Interesting Novel Gene (RING) finger domain protein. In addition to the early flowering time, *FRRP1*-RNAi knockdown transgenic rice caused changes on an array of agronomic traits, including plant height, panicle length and grain length. We analyzed the expression of some key genes associated with the flowering time and other agronomic traits in the *FRRP1*-RNAi knockdown lines and compared with that in wild-type lines. The expression of *Hd3a* increased significantly, which was the key factor in the early flowering time. Further experiments showed that the level of histone H2B monoubiquitination (H2Bub1) was noticeably reduced in the *FRRP1*-RNAi knockdown transgenic rice lines compared with wild-type plants and MBP-*FRRP1*-F1 was capable of self-ubiquitination. The results indicate that Flowering Related RING Protein 1 (*FRRP1*) is involved in histone H2B monoubiquitination and suggest that *FRRP1* functions as an E3 ligase *in vivo* and *in vitro*. In conclusion, *FRRP1* probably regulates flowering time and yield potential in rice by affecting histone H2B monoubiquitination, which leads to changes in gene expression in multiple processes.

## Introduction

The rapid growth in the world population may produce serious food shortages globally in the near future. Consequently, increasing crop grain yields is critical. A staple crop for more than half of the world’s people, rice (*Oryza sativa L*.) has always been deemed important in plant science for its steady yields. Not only are rice yields determined by panicles per plant, grain weight and grain number, but they are also affected by plant height and flowering time (heading date).

The diversity of heading dates is one of the primary reasons for the global cultivation of rice. It is essential for rice to adapt to different cultivation regions and planting seasons, ensuring yield stability through its survival and reproduction [1–3].

Rice is also a model organism for genetic and genomic researches in monocots [4–7]. The transgenic technique is an effective tool for regulating heading date in rice, enabling adaptation to the growth environment.

A number of genes controlling flowering time in rice have been identified and classified. They are distributed mainly in two pathways [8]. Molecular genetic studies have revealed that *Heading date 1* (*Hd1*, a rice ortholog of *Arabidopsis CO*) is regulated by GIGANTEA (*OsGI*, a rice ortholog of *Arabidopsis GI*) and *Hd1* up-regulates *Heading date 3a* (*Hd3a*, a rice ortholog of *Arabidopsis FT*, a major floral activator under SD conditions), thus promoting flowering in short-day (SD) conditions. The entire regulation pathway *OsGI-Hd1-Hd3a* in rice parallels a conserved *GI-CO-FT* pathway in *Arabidopsis* LD activation [9–13]. The long-day (LD) pathway of either activation or suppression of flowering is unique to rice [8,14]. In the LD regulatory pathway, *Hd1* down-regulates *Hd3a* expression and then inhibits flowering in LD conditions [8,15]. Both *Hd3a* and another floral activator *RICE FLOWERING LOCUS T 1* (*RFT1*, a major floral activator under LD conditions) are controlled by *EARLY HEADING DATE 1* (*Ehd1*), which encodes a B-type response regulator [8,15,16]. In addition, *RID1/OsID1/Ehd2*, a homolog of maize (*Zea mays*) *Indeterminate 1*(*ID1*), is necessary for the expression of *Ehd1* regardless of photoperiod [17–19]. *GRAIN NUMBER*, *PLANT HEIGHT AND HEADING DATE 7(Ghd7)*, which encodes a CCT domain protein, is an important regulator of heading date in rice. *Ghd7* represses the expression of *Ehd1* and *Hd3a*, thereby delaying flowering in LD conditions [1]. *DAYS TO HEADING ON CHROMOSOME 8*(*DTH8/Ghd8)*, which encodes a putative HAP3 subunit of the CCAAT-box-binding transcription factor, represses the expression of *Ehd1* and suppresses flowering in LD conditions [7]. As such, all of the flowering regulators that have antagonistic functions eventually converge to control flowering in rice.

Many flowering regulators, such as RID1/OsID1/Ehd2, Ghd7and others, play important roles in influencing many other agronomic traits [1,7,17, 18]. For example, although Ghd7 suppresses flowering in LD conditions it also influences plant height and yields [1,20]. In addition, several quantitative trait loci (QTLs) and rice mutants are known to affect the yield potential, including *QTL for SEED WIDTH ON CHROMOSOME5* (*GW5/qSW5*), *GRAIN SIZE 3* (*GS3*), *GRAIN WEIGHT 2* (*GW2*) and *GRAIN INCOMPLETE FILLING 1* (*GIF1*) [21–26]. Within chromosome segment substitution lines (CSSL) derived from a cross between the IR24 and Asominori parents, CSSL8 and CSSL61, in which the chromosome segment containing *DTH8* from the IR24 is introgressed into the Asominori genetic background, showed earlier heading, and their plant height, number of grains per panicle and dry weight per plant differed significantly from Asominori under natural long day (NLD) conditions [7].

The RING finger domain is a zinc finger domain, first identified in the early 1990s and named for the acronym used for the protein encoded by the *Really Interesting New Gene 1* (*RING*) [27,28]. The majority of RING fingers fall into two subclasses, C3HC4-type RING finger (RING-HC) and C3H2C3-type (RING-H2), depending on whether a histidine or a cysteine residue is in the fifth of the eight Zn coordinating sites [28–30]. Most RING finger proteins are potential ubiquitin ligases.

Protein ubiquitination is achieved through the action of the E1, E2 and E3 ubiquitin ligases [31–34]. There are two main types of protein ubiquitination: multiubiquitination and monoubiquitination. Most of multiubiquitination mark proteins to be degraded by the 26S proteasome, whereas monoubiquitination mediates the localization or activation of the ubiquitinated proteins [35, 36].

Ubiquitination plays an important role in regulating cellular activity by coordinating it with other posttranslational modifications. Numerous proteins are labeled ubiquitin; one of them, histone ubiquitination, is mostly associated with histone H2B and H2A. Ubiquitination of histones is mainly involved in regulating eukaryotic gene expression by altering chromatin structures [37] and controlling the transcription machinery [38,39].

In plants, several RING finger proteins such as E3 ubiquitin ligases have been predicted or known functions in the control of flowering, cell cycle, hormone signaling and stress responses [40–44].

In this study, we cloned a gene, *Flowering Related RING Protein 1 (FRRP1)* (Os10g0565600), that encodes a RING finger protein from rice. Down-regulation transcript of *FRRP1* by RNAi knockdown in transgenic rice plants produced an early flowering phenotype. Interestingly, we also found changes in an array of agronomic traits. Therefore, further researches were performed on the *FRRP1*-RNAi knockdown transgenic rice. The results indicated that the changes in expression of several key genes probably were the reasons for the phenotypes observed.

## Materials and Methods

### Plant Materials and Growth Conditions

We used Nipponbare (*Oryza sativa L*. *japonica*. cv. Nipponbare) as the WT plant in our experiments. Plants for experimental analysis were grown in normal rice growing seasons under natural field conditions in the Shangzhuang Experimental Station of China Agricultural University, Beijing, China. Rice seeds were sown in a seed bed in early May and then transplanted to the field in early June. Field management, including irrigation, fertilizer application and pest control followed usual agricultural practice.

The seeds of *Arabidopsis* used in this study were ecotype Columbia-0 (Col-0). For the mutant complementation assay, the seeds of the *hub2* mutant were used, corresponding to SALK_071289 [45]. For planting, the seeds were kept at 4°C for 3 days, then germinated on Murashige and Skoog (MS) medium. After 6–7 days of growth under continuous white light, the plants were transplanted to soil and grown under conditions with daily cycles of 16 h of light at 22°C and 8 h of dark at 18°C.

### Cloning *FRRP1* and Sequence Analysis

Rice variety Nipponbare were field grown under normal conditions in the greenhouse of China Agriculture University. RNA samples were prepared from leaves. The complementary DNA (cDNA) samples were synthesized using M-MLV reverse transcriptase (Promega, USA). The protocols followed a previous method [46]. The PCR amplification conditions were 95°C for 5 min; 35 cycles of 95°C for 1 min; 55°C for 1 min; and 72°C for 3 min 30 s; with a final extension at 72°C for 10 min, with primers *FRRP1*-F and *FRRP1*-R (S1 Table). The amplified 2535 bp coding sequence (CDS) fragment was sequenced and then inserted into pMD18-T Simple Vector (TaKaRa, Japan) and sequenced. Sequence analysis was performed with DNAMAN (version 6). The phylogenetic tree was constructed in MEGA (version 5.1) using the neighbor-joining method, and bootstrap analysis was performed with 1000 replications.

### Plasmid Construction and Rice Transformation

We had known the HUB2 affected the *Arabidopsis* flowering, so we found FRRP1 in rice database by blast with HUB2 sequence. Then the mutant population were generated by construct RNAi plasmid. To construct the *FRRP1*-RNAi plasmid, a 500-bp fragment of *FRRP1* CDS, only identity with the *FRRP1* CDS in the rice genome, was amplified using primers *FRRP1*-303-F1/*FRRP1*-303-R1 and *FRRP1*-303-F2/*FRRP1*-303-R2 (S1 Table) and inserted into the pTCK303 vector. The final vector pTCK303-*FRRP1* was introduced into Nipponbare via *Agrobacterium tumefaciens* mediated transformation to produce RNAi knockdown transgenic lines. Details of the protocols were as described previously by Wang et al. [47] with a few modifications.

### PCR and Histochemical Analysis of GUS Activity

To determine the integration of the pTCK303-*FRRP1* in the genome, PCR was performed using specific primer pairs (*FRRP1*-Rcheck-F and *FRRP1*-Rcheck-R) (S1 Table) to amplify the specific fragment region from T_0_ and T_3_ generation transgenic rice plants. Genomic DNA (gDNA) was extracted and purified from young leaves using the cetyl trimethyl ammonium bromide (CTAB) method [48]. For histochemical *GUS* analysis, the roots of T_0_ and T_3_ generation transgenic rice samples were analyzed according to the protocol described by Jefferson et al [49].

### Phenotypic Data Collection

The heading date was the day when the first panicle of the plant emerged to a height of 2 cm. The panicle length was the average length of three main panicles measured from the bottom neck to their tips for each plant. Plant height was measured from the ground to the tip of the tallest tiller of the plant. We measured one main panicle three times, from which 10 randomly selected grains were aligned one by one and the average length was recorded as the grain length. A Student’s *t*-test was used in all statistical analyses (P≤0.05).

### Semiquantitative Reverse Transcription PCR and Quantitative Real-Time PCR

RNA samples were prepared from leaves about 1 month before WT plants flowering (expression analysis of key genes for flowering time). The cDNA samples were synthesized by use of M-MLV Reverse Transcriptase (Promega, USA). The protocols followed a previous method [46]. qPCR analysis was performed with a CFX-96 real-time system (Bio-Rad) using SYBR Premix Ex Taq Mix (TaKaRa, Japan). LA *Taq* polymerase (TaKaRa, Japan) was used for semiquantitative RT-PCR with the primers q*FRRP1*-F and q*FRRP1*-R. Several primers from previous papers are listed in S1 Table [45,50,51].

### Genetic Complementation in *Arabidopsis*

For complementation of *Arabidopsis hub2* mutants (SALK_071289), the CDS of *FRRP1* was cloned into the pCAMBIA vector 1305.1 with primers *FRRP1*-1305.1-F and *FRRP1*-1305.1-R (S1 Table), downstream of the CaMV35S promoter. The resultant construct was introduced into *hub2* mutant plants by the floral dip method with *Agrobacterium tumefaciens* (strain EHA105) [45].

### *In Vivo* H2B Monoubiquitination Assay

For the detection of overall H2Bub1 levels between both WT plants and the *FRRP1*-RNAi transgenic rice, total proteins were extracted from 70-day-old plants with plant extraction buffer (0.25 M NaCl, 1% sodium dodecyl sulfate, 1% β-mercaptoethanol, 0.05 M phosphate-buffered saline, pH 7.4, 1 mM phenylmethylsulfonyl fluoride). The proteins were then separated by 10% sodium dodecyl sulfate-polyacrylamide gel electrophoresis and transferred to nitrocellulose membranes for blotting with an antibody against ubiquityl-histone H2B(H2Bub1) monoclonal (Cell Signaling). Histone H3 was used as a loading control and was detected with an anti-histone H3 antibody (Cell Signaling). The protocols followed a previous method [52].

### *In Vitro* Ubiquitination Assay

The reactions system contained 20 ng of purchased E1 (UBE1, rabbit, Boston Biochem), 40 ng of purified E2 (AtUbc1, from *Arabidopsis* saved in our laboratory), 5 μg of purchased *A*. *thaliana* ubiquitin (Boston Biochem) and 3 μg of purified MBP (Maltose Binding Protein)-FRRP1-F1 (*FRRP1-F1* was cloned into pMAL-c2x with an MBP-tag at the N-terminus, expressed in *E*.*coli* TB1 and purified using amylose resin.) in the ubiquitination buffer (0.1 M Tris-HCl pH 7.5, 25 mM MgCl_2_, 2.5 mM dithiothreitol and 10 mM ATP) in a final volume of 30 μL. The reactions were incubated at 30°C for 2 h and then were stopped by the addition of 4×SDS loading buffer. The samples were separated by 10% SDS-PAGE (Polyacrylamide Gel Electrophoresis) gel and detected with anti-MBP and anti-Ubi (mouse monoclonal, Abcam) antibodies by western blotting.

## Results

### Knockdown of *FRRP1* Resulted in Pleiotropic Phenotypes, Especially Early Flowering in Rice

**Fig 1:**
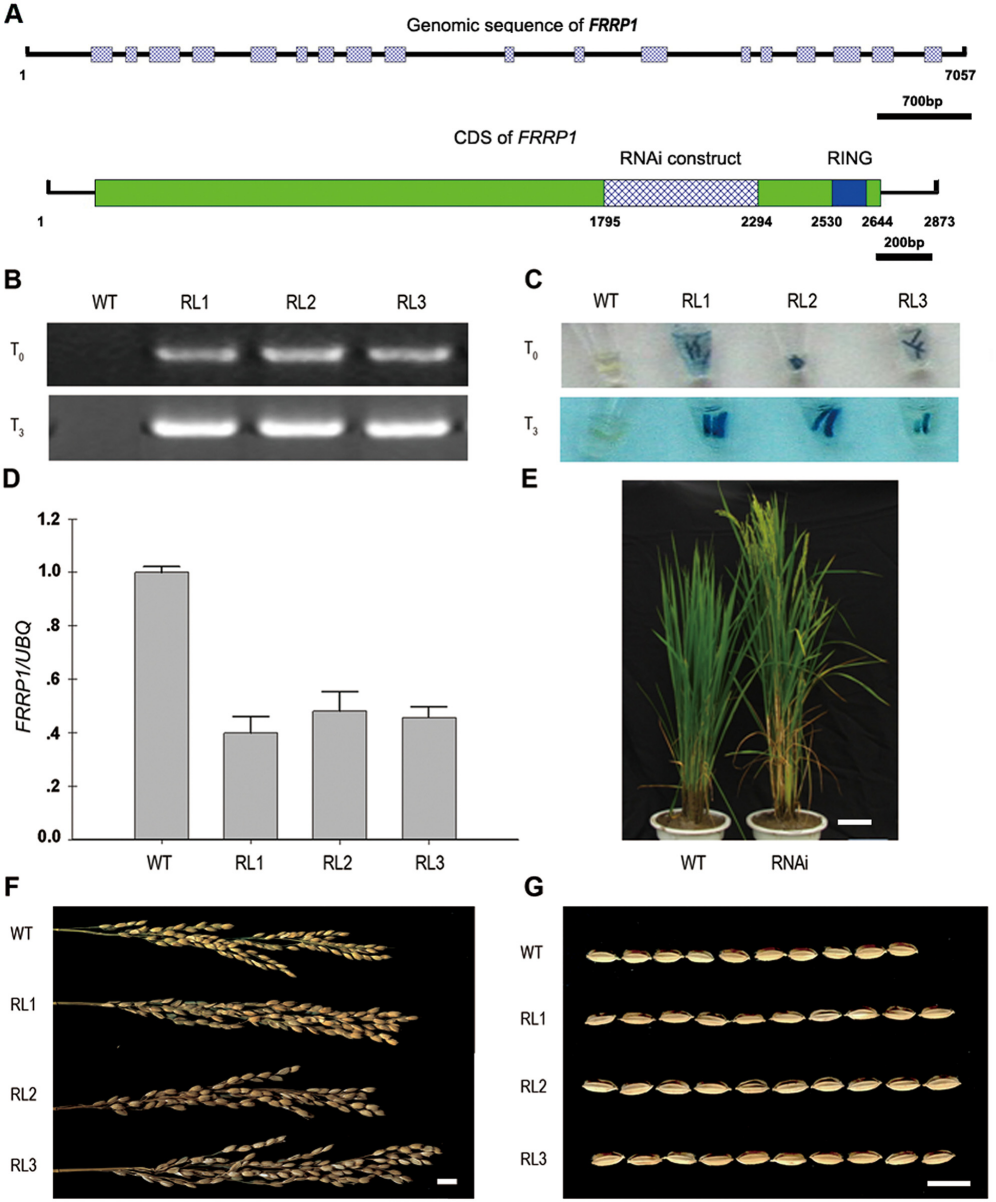
Phenotypes and identification of *FRRP1*-RNAi transgenic rice. (A) Schematic structures of the *FRRP1* genomic sequence (upper line) and cDNA clone (lower line). The hatched bar represents the exons, the solid lines depicts the introns (upper line), the green solid bar represents the coding region, the spotted bar shows the region targeted for RNAi, and the blue solid bar depicts the RING domain containing 39 amino acids at the C-terminus (lower line). (B) The presence of the *FRRP1*-RNAi transgene in T_0_ (top) and T_3_ (bottom) generations of the *FRRP1*-RNAi transgenic rice (RL1, RL2 and RL3) compared with WT plants by PCR analysis. (C) Integration of *FRRP1* into the genome of the transgenic rice. The expression of GUS fused in the vector pTCK303-*FRRP1* in the radicle of T_0_ (top) and T_3_ (bottom) generations of the *FRRP1*-RNAi transgenic rice (RL1, RL2 and RL3) compared with WT plants by GUS histochemical staining. (D) Reduced expression of *FRRP1* in *FRRP1*-RNAi transgenic seedlings (RL1, RL2 and RL3) compared with the expression in WT plants. *Ubiqutin* was used as an internal control. Error bars represent ± SD. (E). *FRRP1*-RNAi transgenic rice showed an early flowering phenotype. Several panicles emerged in the transgenic rice (RNAi) but none in WT plants. Rice plants were photographed at 105 days after sowing. The unit of scale bar was 10 centimeters (cm). (F). *FRRP1*-RNAi transgenic plants (RL1, RL2 and RL3) showed longer panicles than that of WT plants, photographed after harvesting. The unit of scale bar was 1 centimeter (cm). (G) *FRRP1*-RNAi transgenic plants (RL1, RL2 and RL3) showed longer grain length compared with the length of WT plants, photographed after harvesting. The unit of scale bar was 1 centimeter (cm).

To obtain further knowledge of floral induction in rice, we used RNAi to produce transgenic plants and measured flowering time in each line. We found that one of the transgenic plants showed significantly early flowering time compared with wild type (Nipponbare). We cloned the full-length cDNA of the gene disrupted in this RNAi line and found it comprised of 2873 base pairs (bp). Its open reading frame (ORF) was 2535 bp, corresponding to an 844-amino-acid polypeptide with a C3HC4-type RING in the C-terminal. It had a predicted molecular mass of 96.5 kilodaltons (kDa) and calculated isoelectric point (pI) of 4.90 (Fig 1A). We named the protein Flowering Related RING Protein 1 (FRRP1) (Os10g0565600).

Thirteen T_0_ transgenic *FRRP1*-RNAi rice lines were produced after several rounds of selection by polymerase chain reaction (PCR) and β-glucuronidase (GUS) staining (Fig 1B and 1C). Three independent lines randomly selected from the T_3_ populations derived from these lines were labeled RL1, RL2 and RL3 and used for phenotypic analysis. Further quantitative reverse transcription (qRT)-PCR analysis of *FRRP1*-RNAi transgenic plants confirmed that expression of *FRRP1* in the transgenic lines RL1, RL2 and RL3 were reduced to 40%, 48% and 46% of the wild-type (WT) level, respectively (Fig 1D), establishing that the knockdown of *FRRP1* were implemented successfully in the selected transgenic lines.

To analyze the total days to heading, seeds from the T_3_ generations were planted in natural long-day (NLD) conditions (day length >14 h). Nipponbare was the wild type used as a control. Compared with the 118.5 days to heading for WT plants, the three transgenic knockdown lines flowered 23–26 days earlier under the same growth conditions (Fig 1E, Table 1). This suggested that knockdown of *FRRP1* significantly accelerated flowering in rice under NLD condition. In addition, *FRRP1*-RNAi knockdown transgenic plants exhibited other phenotypic traits: their plant height were 10–20 cm higher than that of WT plants, and the grain length were 0.06–0.08 cm longer compared with that of WT plants. Also, panicle length showed a change to some extent in NLD conditions, and two lines were 2cm longer than that of WT plants (Fig 1F and 1G, Table 2)

**Table 1:** Flowering times of WT and *FRRP1*-RNAi transgenic plant*s in rice*.

Line	No.	Flowering time (day)
WT	15	119 ± 2
RL1	15	96 ± 2**
RL2	15	93 ± 2**
RL3	15	94 ± 2**

Values are means ± standard deviation (*N* = 15).

**Extremely significant difference compared with WT (*P* ≤ 0.01).

**Table 2:** Values of other agronomic traits in WT and *FRRP1*-RNAi transgenic plants in rice.

Line	Plant height (cm)	Panicle length (cm)	Grain length (cm)
WT	93±4	18±1	0.74±0.011
RL1	113±5**	20±1**	0.80±0.020**
RL2	103±4**	19±1	0.81±0.023**
RL3	104±5**	20±1**	0.82±0.033**

Values are means ± standard deviation (N = 15).

**Significant difference compared with WT (P ≤ 0.01).

### Analysis of *FRRP1*

**Fig 2:**
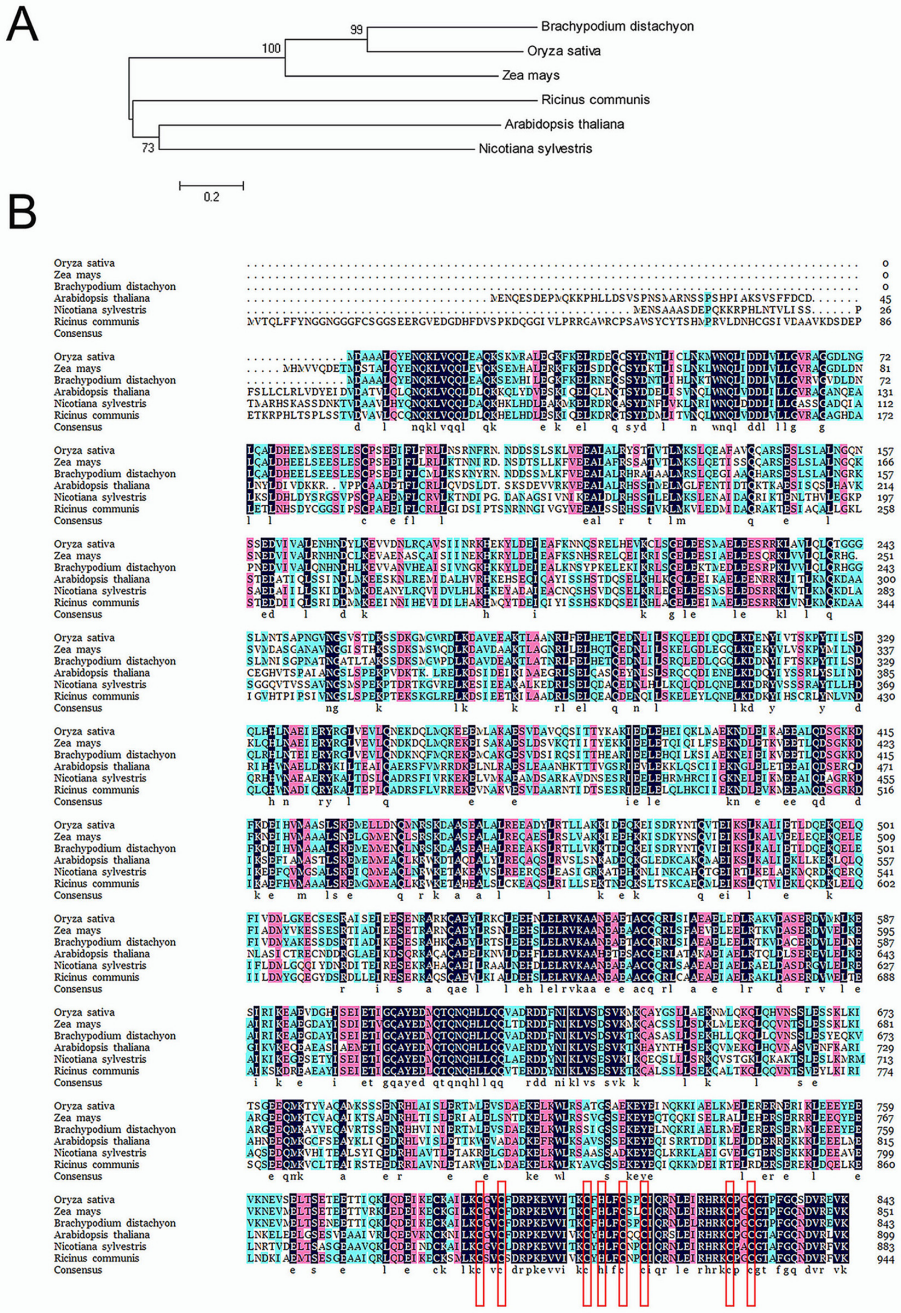
Sequence analysis of *FRRP1*. (A) Phylogenetic relationship between FRRP1 and five other FRRP1orthologs from *Zea*, *Brachypodium*, *Arabidopsis*, and *Nicotiana* and *Ricinus*. The phylogenetic tree was constructed using the neighbor-joining method in the MEGA software package (version 5.1) and bootstrap analysis was performed with 1000 replications. The scale bars represent the number of substitutions per site. (B) Multiple sequence alignment of FRRP1 and closely related proteins from different species. The derived amino acid sequence of FRRP1 is compared with the proteins that showed high identity with FRRP1 from five species. The corresponding proteins are an E3 ubiquitin-protein ligase BRE1-like protein (XP_003568771.1) from *Brachypodium distachyon*, a RING domain protein (XP_008668811.1) from *Zea mays*, a RING domain protein (GenBank accession number XP_002530869.1) from *Ricinus communis*, histone monoubiquitination 2 (AtHUB2) (At1g55250) from *Arabidopsis thaliana* and a RING domain protein (GenBank accession number XP_009801022.1) in *Nicotiana sylvestris*. Eight amino acid residues conserved in and characteristic of the RING domain proteins are framed in red, and these amino acid residues were identical in all six proteins.

A database search and multiple sequence alignment showed that FRRP1 (Os10g0565600) shared 50% identity with *Arabidopsis thaliana* histone monoubiquitination 2 (AtHUB2) (At1g55250), 53% identity with the homolog of *Nicotiana sylvestris* (GenBank accession number: XP_009801022.1) and 55% identity with that of *Ricinus communis* (GenBank accession number: XP_002530869.1). On the one hand, FRRP1 shared a significant (80%) sequence identity with an E3 ubiquitin-protein ligase BRE1-like protein (GenBank accession number XP_003568771.1) in *Brachypodium distachyon*. On the other hand FRRP1 revealed a considerable (76%) sequence identity with a corresponding protein (GenBank accession number XP_008668811.1) in *Zea mays* (Fig 2B). All of these relevant proteins contain a single C3HC4-type RING domain in their C-terminal regions. Fig 2A shows the phylogenetic relationship among FRRP1 from *Oryza sativa* and five other orthologs from *Brachypodium*, *Zea mays*, *Ricinus*, *Arabidopsis* and *Nicotiana*. It suggests that RING domain was conserved in monocotyledonous, but it was different in dicotyledonous plants. The RING domain of FRRP1 showed a very high amino acid sequence identity (90%–97%) with the conserved plant RING finger domains in the approximately 45-amino-acid RING motifs. All of the aforementioned proteins contain Cys-X2-Cys-X(9–39)-Cys-X(1–3)-His-X(2–3)-Cys-X2-Cys-X(4–48)-Cys-X2-Cys, which is the basic sequence of the canonical RING domain (Fig 2B).

### *FRRP1* Complements the *Arabidopsis hub2* Mutant

**Fig 3:**
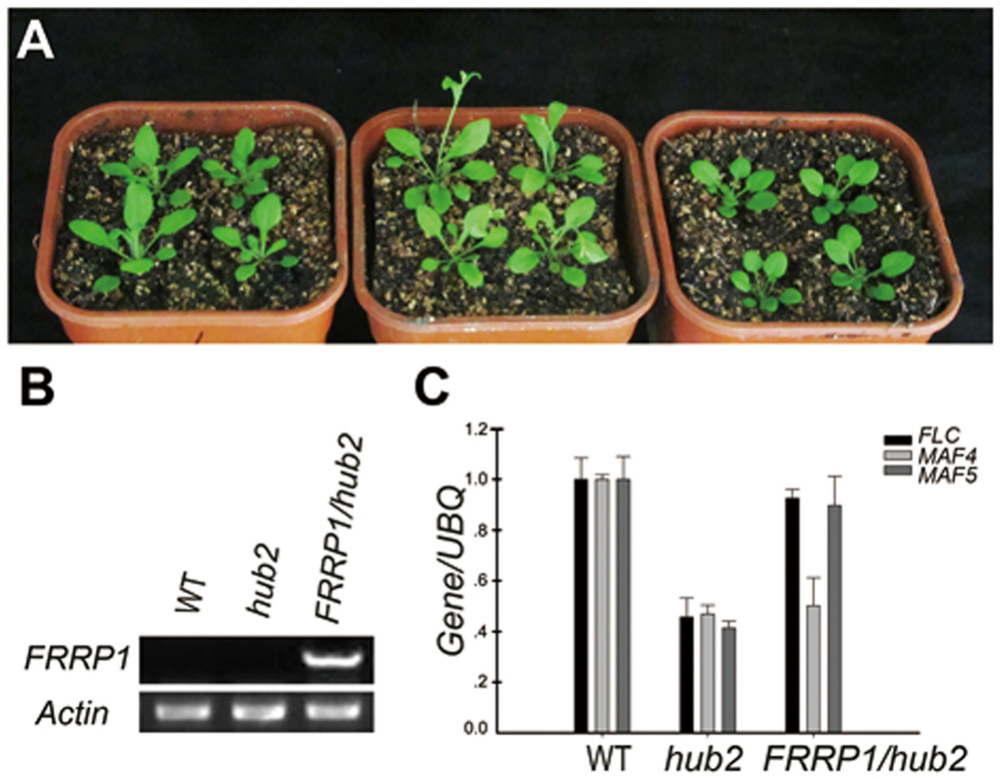
Complementation analysis of *FRRP1* in *Arabidopsis*. (A) Phenotype of flowering time in *Arabidopsis*. When *hub2* mutant plants (middle) flowered, the complemented *hub2* plants (right) did not flower as WT (Col-0) plants (left) (B) Semi-qRT-PCR analysis of *FRRP1* in *Arabidopsis*. *Actin* was used as an internal control. (C). Expression of *FLC* clade genes in *Arabidopsis*. Expression of *FLC* and *MAF5* was restored to near WT levels in *hub2* plants stably transformed with *FRRP1*.

Most RING finger proteins were E3 ligases [53], and FRRP1 was 50% identical with AtHUB2 (At1g55250), an E3 ligase in *Arabidopsis* regulating flowering time and involved in H2Bub1(On the histone H2B plus a ubiquitin protein, such as transcription regulation process, not the degradation of protein) [45]. To determine how FRRP1 regulated flowering way, a genetic complementation assay was performed by transforming *FRRP1* under control of a constitutive CaMV 35S promoter into *Arabidopsis hub2* mutant plants [45]. We obtained nine *35S*:*FRRP1*/*hub2* plants lines, three of which were complemented. The T_3_ generation homozygotes of transgenic *hub2* harboring *FRRP1* were used for observation and analysis. The flowering time of the transgenic *hub2* mutant was complemented by rice FRRP1 (Fig 3A and 3B, Table 3). Furthermore, qPCR analysis of the *FLC*-clade genes demonstrated that expression of *FLC* and *MAF5* in the transgenic *hub2* plants can be restored to the level in WT plants (Fig 3C). FLC inhibits the expression of flowering time integrators, including *FLOWERING LOCUS T* (*FT*) and *SUPPRESSOR OF OVEREXPRESSION OF CONSTANS1*, and it indirectly represses such floral meristem identity genes such as *LEAFY* and *APETALA1* [54–56]. There are five *FLC* homologs, *MADS AFFECTING FLOWERING* (*MAFs*), in the *Arabidopsis* genome, each of which is also involved in the control of flowering time [57–59]. These results suggested that FRRP1 plays a similar role as AtHUB2 in control of flowering time in *Arabidopsis*.

**Table 3:** Leaf number at flowering of the wild type (Col-0), *hub2* mutant and the complemented *hub2* mutant.

Genotype	No. of rosette leaves
WT(Col-0)	10 ± 1
*hub2*	8 ± 1*
*FRRP1/hub2*	10 ± 1

*Significant difference compared with WT (Col-0) (*P* ≤ 0.05).

Values are means ± standard deviation (*N* = 15).

### The Reduced Monoubiquitination of Histone H2B in the *FRRP1*-RNAi Knockdown Transgenic Lines

**Fig 4:**
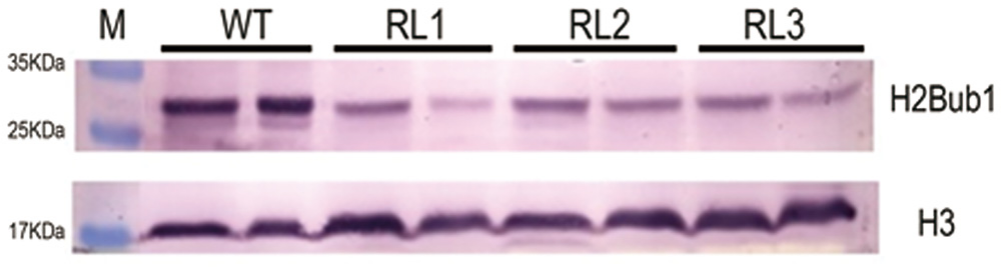
Monoubiquitination of histone H2B *in vivo*. Detection of H2Bub1 in *FRRP1*-RNAi transgenic lines (RL1, RL2 and RL3) and WT, using a monoclonal antibody against H2Bub1. The H2Bub1 level was lower in *FRRP1*-RNAi transgenic lines (RL1, RL2 and RL3) than in WT plants. Every line has two biological replicates. Histone H3 was used as a loading control.

To verify whether FRRP1 affects gene expression through its E3 ligase activity, we extracted total proteins from the *FRRP1*-RNAi transgenic lines and used the monoclonal antibody against ubiquity1-histone H2B(H2Bub1) to detect the monoubiquitinated histone H2B in the transgenic rice plants, using histone H3 as a loading control. The H2Bub1 level was significantly reduced in the *FRRP1*-RNAi knockdown transgenic lines compared with that of WT plants (Fig 4). This suggested that the FRRP1 functioned as an E3 ligase and monoubiquitinated histone H2B *in vivo*.

### *FRRP1*-F1 Have the Ability of Self-Ubiquitination *In Vitro*

**Fig 5:**
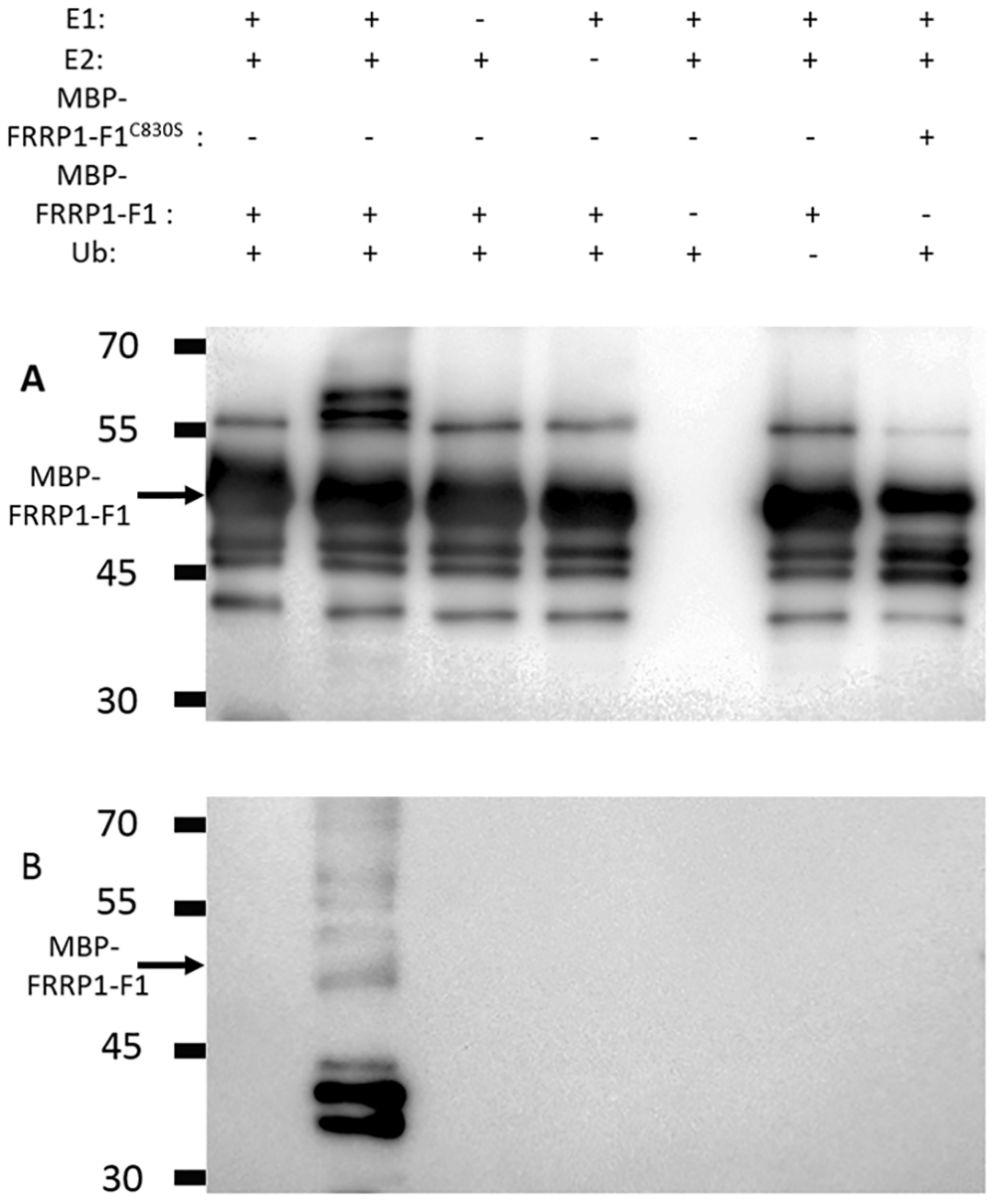
Ubiquitin ligase activity of *FRRP1*-F1(*FRRP1*). The MBP-FRRP1-F1 and MBP-FRRP1-F1^C830S^ fusion protein were assayed for ubiquitin activity in the presence or not presence of E1, E2 and/or ubiquitin. The numbers on the left denoted the molecular mass of marker proteins in kiloDaltons. (A) The anti-MBP antibody was used to detected maltose-fusion proteins (upper panel). (B) The anti-Ub antibody was used to detect ubiquitin (lower panel).

To get more evidences that FRRP1 is an E3 ligase, a ubiquitination test was performed *in vitro*. Because we couldn’t obtain the full length and soluble protein of FRRP1, we created a truncated protein, named FRRP1-F1. It was the C-terminal region of FRRP1 (amino acid residues 786 to 844). In order to find out the key amino acid involved in the function of E3 ligase activity in the RING domain, Cys^830^ was substituted with Ser^830^ in the FRRP1-F1, named FRRP1-F1^C830S^. Then FRRP1-F1 and FRRP1-F1^C830S^ were fused with a maltose-binding protein (MBP-FRRP1-F1 and MBP-FRRP1-F1^C830S^), expressed in *E*.*coli* TB1, and purified using amylose resin. The ubiquitinated protein MBP-FRRP1-F1 of high molecular weight was detected at the presence of E1 (ubiquitin-activating enzyme), E2 (ubiquitin-conjugating enzymes), ubiquitin and ATP, suggesting that MBP-FRRP1-F1 was capable of self-ubiquitination (Fig 5A and 5B, lane 2). But the mutant MBP-FRRP1-F1^C830S^ did not have the ability of self-ubiquitination (Fig 5A and 5B lane 7). The *in vitro* ubiquitination tests identified FRRP1-F1(FRRP1) as E3 ligase. However, FRRP1-F1^C830S^ had lost the E3 ligase activity *in vitro*. And the results of ubiquitination reaction assay also showed that the amino acid Cys^830^ in the RING domain was required for the activity of FRRP1-F1(FRRP1).

### The Expression Profiling of Key Genes for Flowering Time and Other Traits in the Knockdown Transgenic Rice Lines

**Fig 6:**
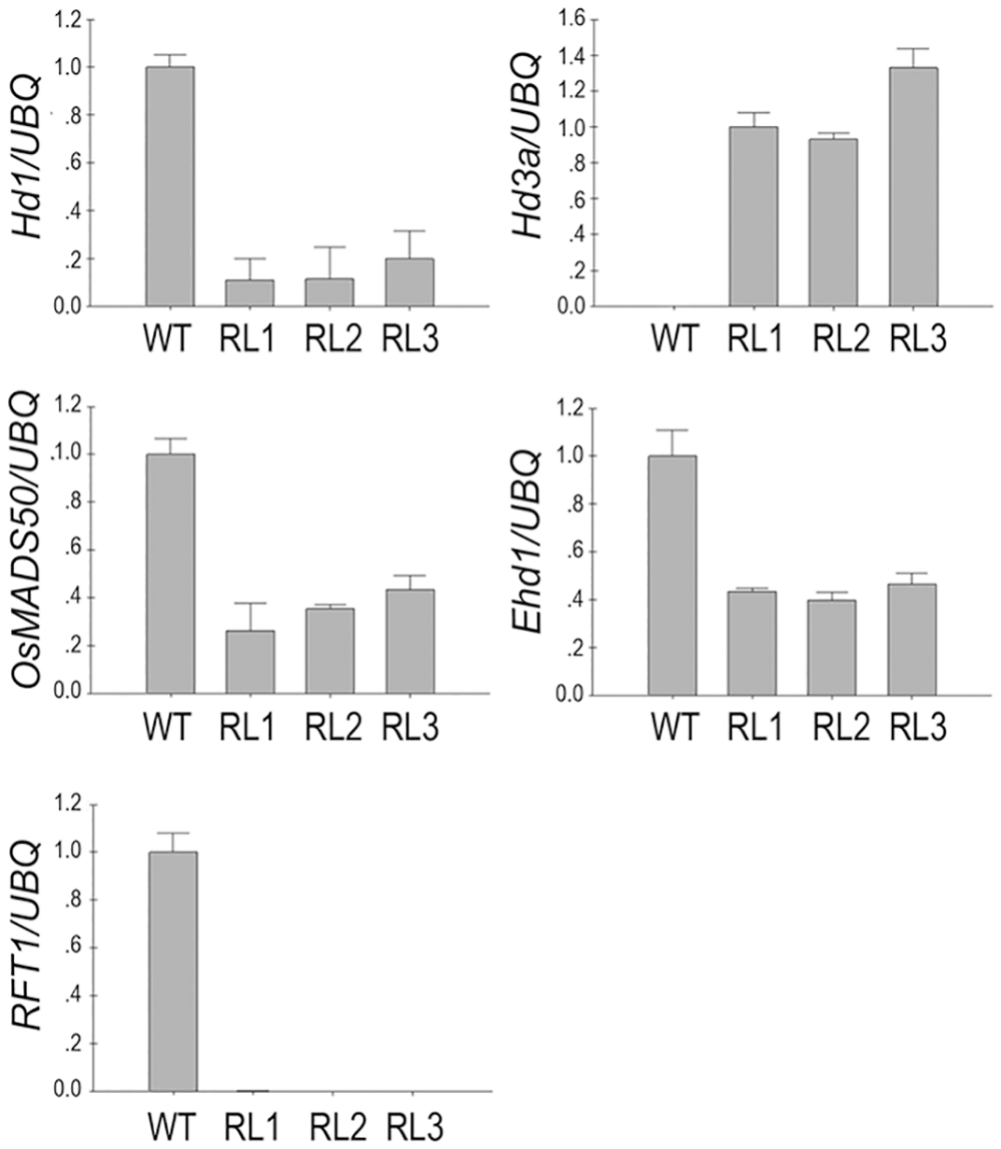
Expression analysis of genes associated with flowering time. *Expression of OsMADS50*, *Hd1*, *Ehd1*, *RFT1 and Hd3a was measured using quantitative RT-PCR*. The expression of *RFT1* was almost undetected in *FRRP1*-RNAi transgenic lines (RL1, RL2 and RL3), whereas *Hd3a* was undetected in the WT plants. These experiments were repeated at least three times. *Ubiquitin* was used as an internal control. Error bars represent ± SD.

To explain the molecular mechanism affecting the phenotypic alteration in the transgenic rice, expression of several key genes was evaluated, such as *OsMADS50* [60], *Hd1*, *Ehd1*, *Hd3a* and *RFT1*. The *FRRP1*-RNAi knockdown transgenic lines and the WT plants were grown under natural field conditions(NLD condition) (day length >14h). About 30 days before rice flowering, we collected samples for analysis. The expression of *OsMADS50*, *Hd1* and *Ehd1* was reduced and *RFT1* was barely detectable in the transgenic rice compared to that in WT plants. By contrast, the transcript of *Hd3a* was greatly increased in the transgenic lines, whereas it was almost undetectable in the WT plants (Fig 6). These results showed that it was the final result that early flowering in the *FRRP1*-RNAi knockdown transgenic lines was due to the altered expression of a combination of genes regulating flowering. Upregulation of Hd3a corresponded with earlier flowering in the transgenic lines.

**Fig 7:**
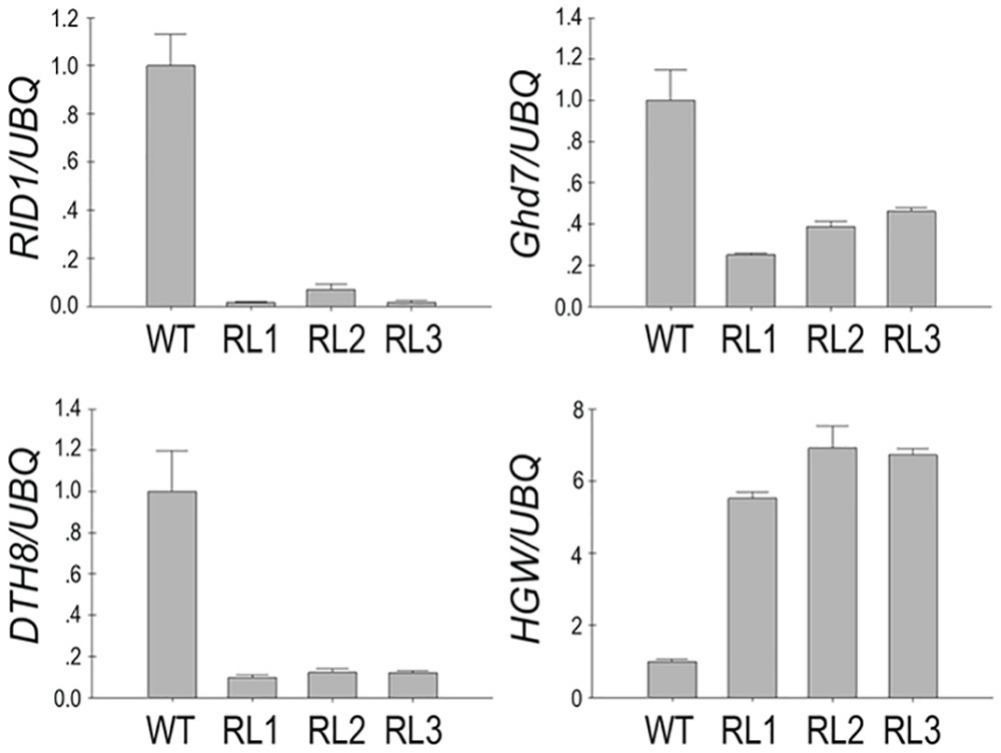
Expression analysis of genes associated with both flowering time and yield. *Expression of DTH8*, *RID1*, *HGW* and *Ghd7was measured using quantitative RT-PCR*. These experiments were repeated at least three times. *Ubiquitin* was used as an internal control. Error bars represent ± SD.

We also examined expression of a cluster of genes (*RID1/OsID1/Ehd2*, *Ghd7*, *DTH8/Ghd8* and *HGW*) that controlled both flowering time and other agronomic traits [3]. The transgenic lines all showed reductions in mRNA levels of these genes, except *HGW*, whose expression increased (Fig 7). Possibly the changes of the expression of the aforementioned genes varied the phenotypes of the transgenic plants.

**Fig 8:**
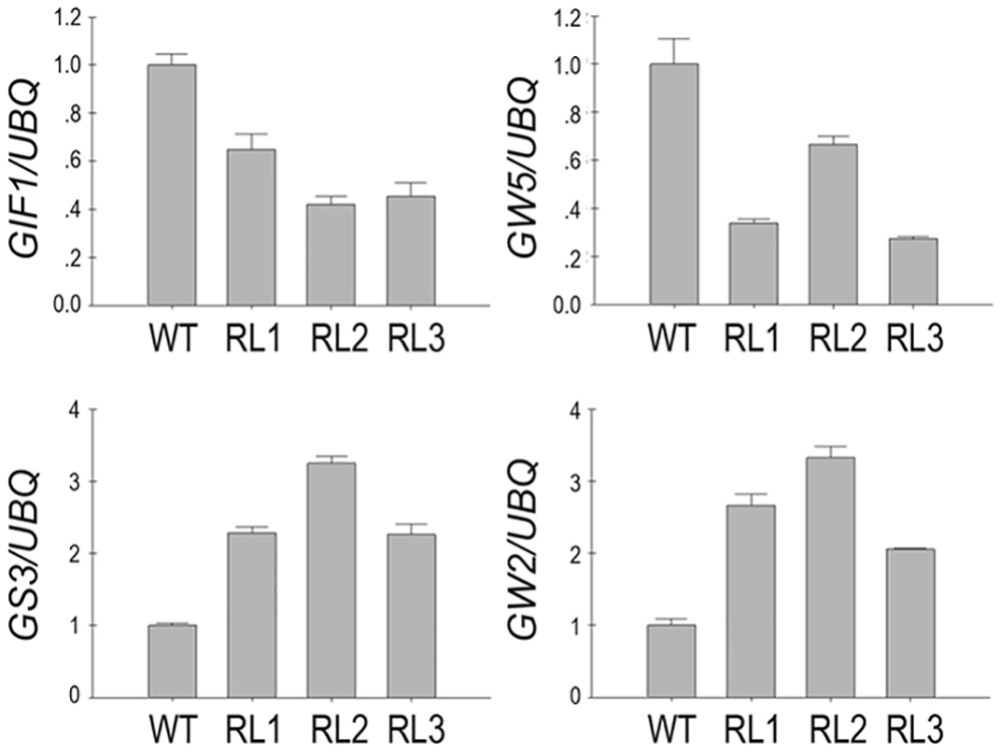
Expression analysis of genes associated with grain shape and yield. *Expression of GS3*, *GIF1*, *GW2* and *GW5 was measured using quantitative RT-PCR*. These experiments were repeated at least three times. *Ubiquitin* was used as an internal control. Error bars represent ± SD.

After examining the known flowering-related genes, we further investigated whether *FRRP1* acts through *GIF1*, *GW2*, *GW5* and *GS3* to regulate grain size and weight. The results showed the expression of *GW2* and *GS3* was up-regulated, whereas expression of *GW5* and *GIF1* was down-regulated (Fig 8), suggesting that *FRRP1* also affected the expression of grain-related genes in transgenic rice.

From all of the results, we concluded that the most important and direct reason for the significant early flowering of transgenic rice plants was the marked increase in the expression of *Hd3a*. The trait changes in transgenic rice may be regulated collectively by a series of genes involved in controlling flowering time and yield potential, as all of them were possibly affected via *FRRP1*.

## Discussion

### Basic Function of the RING Proteins such as *FRRP1* Is Conserved between Dicotyledonous and Monocotyledonous Plants

Here we performed characterization and analysis of FRRP1, a RING finger protein from rice. Sequence alignment showed that FRRP1 was 50%–80% identical with some other RING finger proteins in different plant species (Fig 2B), and the RING domains featured a more significant sequence identity (~90%–97%). The high level of identity of these RING finger proteins from different plant species suggested that they were highly conserved across both dicotyledonous and monocotyledonous plants. A detailed analysis obtained from the phylogenetic tree is shown in Fig 2A.

AtHUB2 is a RING finger protein and an E3 ubiquitin ligase. A defect in H2Bub1 in the *hub2* mutant of *Arabidopsis* inhibited expression of genes in the *FLC*-clade and thus accelerated the transition to flowering in the *hub2* mutant of *Arabidopsis* [41,44,45]. H2Bub1 levels were reduced in the *FRRP1*-RNAi knockdown rice lines used in our experiments (Fig 4). At the same time, transformants of *Arabidopsis hub2* plants harboring overexpressing *FRRP1* flowered at the same time as WT plants (Fig 3A). Furthermore, there was no difference in the transcript levels of *FLC* and *MAF5* between the transformants and WT plants, although the expression of *MAF4* was inhibited in the transformants (Fig 3C). Such an inconsistency of these three genes in *35S*:*FRRP1*/*hub2* plants and WT *Arabidopsis* may reflect functional divergence between dicotyledonous and monocotyledonous orthologs. In support of this claim, the *Arabidopsis hub2* mutant showed dwarfism [45], but *FRRP1*-RNAi transgenic rice lines were taller than the WT (Fig 1E). These observations suggested a combination of conserved and divergent functions of FRRP1 compared with AtHUB2.

### Increased *Hd3a* Transcript Level Accounts for Early Flowering of *FRRP1* Knockdown Transgenic Rice under NLD Conditions

Knowledge obtained from the considerable efforts made to date in studies of flowering-time regulation has facilitated an understanding of molecular mechanisms controlling heading date in rice. *Hd3a* (a major floral activator under SD conditions) and *RFT1* (a major floral activator under LD conditions) were the most important floral activators, as mobile flowering signals acting to promote the floral transition [8,12,15]. Short days activate the pathway leading to flowering in rice. *Hd3a* is activated by *Hd1*, and also by *Ehd1*, which encodes a B-type response regulator and functions independently of *Hd1* under SD conditions [10,14,16]. Furthermore, *RFT1* was activated later during the development of SD flowering in *Hd3a* RNAi plants [15]. However, Under LD conditions, flowering time is regulated by both activation and suppression pathways interacting in a network. *Hd1* suppresses *Hd3a* expression under LD conditions [14, 15], and *RFT1*, with its both positive (*OsMADS50* and *Ehd1*) and negative (*Hd1* and *Ghd7*) regulators form a gene network to control LD flowering in rice [8].

We constructed *FRRP1*-RNAi knockdown transgenic rice, and found the transgenic rice plants displayed a significant phenotype of early flowering. We investigated the expression of the genes related to flowering time in transgenic rice and the WT plants grown under the same natural field in NLD conditions (Figs 6 and 7). Our qRT-PCR results revealed that the mRNA expression of *OsMADS50*, *Ghd7*, *DTH8*, *RID1/OsID1/Ehd2*, *Ehd1*, *Hd1* and *RFT1* was reduced in the *FRRP1*-RNAi knockdown transgenic rice lines compared with the expression of these genes in WT plants, but transcription of *Hd3a* increased significantly. According to previous studies, under LD conditions, *Hd3a* and *RFT1* were both suppressed by *Hd1* and *Ghd7*, but *RFT1* was promoted by *OsMADS50* and *Ehd1*. Furthermore other studies indicated *Ehd1* was up-regulated by *OsMADS50* and *RID1/OsID1/Ehd2* but suppressed by *Hd1*, *Ghd7* and *DTH8* under LD conditions. Hence all of the regulation mechanisms involved with *Ehd1* may be independent of each other, and indicated that *Ehd1* integrated multiple pathways in the regulation of flowering time [1,7,8]. We conjectured that the genes worked concertedly on *Ehd1*, which resulted in a significant decrease of the *Ehd1* transcript and in the reduced expression of *OsMADS50*, which together decreased the expression of *RFT1*. But the lessened expression of *Hd1* and *Ghd7* imparted a significant increase to the *Hd3a* expression as shown in our results (Fig 6). Finally, an early flowering time in our knockdown transgenic rice was observed (Fig 1E, Table 1). The results indicated that there was a net genetic regulation promoted NLD flowering via *Hd3a* but not *RFT1* in control of multiple genes.

### Pleiotropic Effects of *FRRP1* May Be a Result of a Reduction in H2Bub1 Level

A number of genes involved in controlling heading date, grain size or plant height have been reported recently [1,3,7, 61]. *Ghd7* has been proven to have pleiotropic effects on flowering time, plant height and on rice cultivars adapting to cold climate regions [1]. *DTH8* not only delayed rice flowering but also played an important role in regulating plant height and grain number [7].

Besides early flowering time, *FRRP1*-RNAi knockdown transgenic rice displayed an array of phenotypes, such as higher plant height and longer grain length. These implied *FRRP1* was important for rice development. Therefore the *FRRP1* and *FRRP1*-RNAi knockdown transgenic rice could be used to breed superior rice through genetic engineering and/or conventional breeding.

Up to now, posttranslational modifications of histone, such as acetylation, methylation, phosphorylation and monoubiquitination have been associated with control of several complex processes including plant development and adaptation to environmental conditions [62–64]. Histone H2B modification by monoubiquitination is mainly involved in transcriptional activation and plays a key role in the plant life cycle, such as seed germination and initiation of flowering [65]. For instance, the monoubiquitination of histone H2B was proven to be essential for the enhancement of H3K4 and H3K36 hypermethylation and *FLC/MAF* transcriptional activation in *Arabidopsis*, the deficiency of which leads to earlier transition to flowering time in the *hub2* mutant [43,44,45].

As changes in transcripts of a number of genes were associated with displayed phenotypes of flowering time and yield potential in the *FRRP1* knockdown transgenic rice (Figs 6–8; Tables 1 and 2), the underlying reason was probably the reduction in H2Bub1 level. This may trigger alteration of expression in certain master genes, then cause a series of changes in their regulated pathways and finally produce discernible pleiotropic effects of the phenotypes. In addition, the MBP-FRRP1-F1 had the ability of self-ubiquitination *in vitro*(Fig 5). Thus, we argue that FRRP1 regulated flowering time, plant height and yield potential by monoubiquitinating histone H2B; however, more evidence is required to confirm this hypothesis.

## Conclusion

We obtained several *FRRP1*-RNAi knockdown transgenic rice lines and the rice showed an early flowering and high plant, long grain phenotypes. The transgenic lines flowered much earlier, 23–26 days before the WT rice plants. The phenotype of flowering time was excellent in transgenic rice. To understand the reason and determine the function of FRRP1, we performed qRT-PCR and verified lower levels of histone H2B monoubiquitination of the transgenic rice. And *in vitro* we confirmed the MBP-FRRP1-F1(FRRP1) had the ability of self-ubiquitination. In addition, we performed some tests in *Arabidopsis*. All of the results showed the increase in levels of *Hd3a* was the main reason for the rice early flowering, and many other genes such as *Ehd1*, *Hd1*, *Ghd7* formed a network regulating flowering time and other agronomic traits. H2Bub1 decreased in the transgenic rice and the ability of self-ubiquitination with MBP-FRRP1-F1(FRRP1) *in vitro*, which suggests that FRRP1 was an E3 ligase *in vivo* and *in vitro*, regulating flowering time, plant height and grain length.

## Supporting Information

S1 Table Primers used in the experiments. (DOC)

S2 Table Values of several agronomic traits between transgenic lines and WT in rice. (DOCX)
