# Supplementary material for: Flowering-Related RING Protein 1 (FRRP1) Regulates Flowering Time and Yield Potential by Affecting Histone H2B Monoubiquitination in Rice (Oryza Sativa)
Source: PLoS One. 2016 Mar 2;11(3):e0150458. doi: 10.1371/journal.pone.0150458 (PMC4774988; doi:10.1371/journal.pone.0150458)
Supplement: S1 Table — (DOC) [file pone.0150458.s001.doc]

**S1 Table. Primers used in the experiments.**

| Primer name | **5’-3’** |
| --- | --- |
| Primers for Cloning *FRRP1*, constructs and screening | |
| *FRRP1-*F | ATGGATGCCG CAGCTCTTCA |
| *FRRP1-*R | TCAGATCTTC ACCTCCCGAA |
| *FRRP1-*303-F1 | ATGGATCCAAAAGCAGCAAATGAAGCGG |
| *FRRP1-*303-R1 | ATGGTACCCCTTCTCTGCATCTGATACC |
| *FRRP1-*303-F2 | ATGAGCTCAAAAGCAGCAAATGAAGCGG |
| *FRRP1-*303-R2 | ATACTAGTCCTTCTCTGCATCTGATACC |
| *FRRP1-*Rcheck-F | ATGAGCTCAAAAGCAGCAAATGAAGCGG |
| *FRRP1-*Rcheck-R | TGAAAATCTCGAAACAGCCGTGTCATAGTC |
| *FRRP1-*1305.1-F | ATCCATGGTAATGGATGCCGCAGCTCTTCA |
| *FRRP1-*1305.1-R | ATACTAGTTCAGATCTTCACCTCCCGAA |
| Primers for expression qPCR analysis | |
| Semi-quantitative reverse transcription PCR and quantitative real-time PCR | |
| q*FRRP1-*F | TGGAGCTAGAGCGTGAGAGA |
| q*FRRP1-*R | ATGGCGAGCAGAATAAGTGG |
| *Ubq*-F | AACCAGCTGAGGCCCAAGA |
| *Ubq*-R | ACGATTGATTTAACCAGTCCATGA |
| *Ehd1*-F | TGCAAATGGCGCTTTTGAT |
| *Ehd1*-R | ATATGTGCTGCCAAATGTTGCT |
| *RFT1*-F | TGACCTAGATTCAAAGTCTAATCCTT |
| *RFT1*-R | TGCCGGCCATGTCAAATTAATAAC |
| *Hd3a*-F | GCTCACTATCATCATCCAGCATG |
| *Hd3a*-R | CCTTGCTCAGCTATTTAATTGCATAA |
| *Hd1*-F | TCAGCAACAGCATATCTTTCTCATCA |
| *Hd1*-R | TCTGGAATTTGGCATATCTATCACC |
| *Ghd7*-F | ATGGGGATGGCCAATGAGGAGTC |
| *Ghd7*-R | GAGGAATCCGGCCGCCTTTTTTC |
| *OsMADS50*-F | CAGGCCAGGAATAAGCTGGAT |
| *OsMADS50*-R | TTAGGATGGTTTGGTGTCATTGC |
| *RID1/OsID1/Ehd2*-F | CGACGACAATAGCTCGATCGC |
| *RID1/OsID1/Ehd2*-R | GTGCATGGTCACGGAGCCTT |
| *DTH8*-F | CAGGAGTGCGTGTCGGAGTT |
| *DTH8*-R | GGTCGTCGCCGTTGATGGT |
| *HGW*-F | ACAACTCCCACTACTTCTGTGGCT |
| *HGW*-R | TTGAGGATGTGAAGCCCATCTCGT |
| *GW2*-F | CTGCCTTTCGCCGAGAACTTC |
| *GW2*-R | GCTCTACCTACAACCATGCCAAC |
| *GW5*-F | AGGTGGTGGTGGTGGAGTCC |
| *GW5*-R | GCGTGGCGGTCGTTCTCG |
| *GIF1*-F | TGCATGATGAGAACTACCTTCAG |
| *GIF1*-R | ACTGAAACCATTTTACACAAGGG |
| *GS3*-F | CAGCGACGGCAGCAGCAG |
| *GS3*-R | CATCCTCCTCCTCCTCCTCCTTC |
| FRRP1 complements Arabidopsis *hub2* mutant | |
| *FLC-*F | CCTCTCGTGACTAGAGCCAAG |
| *FLC-*R | AGGTGACATCTCCATCTCAGCTTC |
| *MAF4*-F | GCTTCTCAAGTAACCACCATCAC |
| *MAF4*-R | CTTGGATGACTTTTCCGTAGCAG |
| *MAF5*-F | CATGGATTGTGCTAGAAAACAACTG |
| *MAF5*-R | GCTTCACTCTTCCGACACATCTAATC |
| *ACTIN-F* | GGTGTCATGGTTGGTATGGGTC |
| *ACTIN-R* | CCTCTGTGAGTAGAACTGGGTGC |
| Primers for Expression FRRP1-F1 | |
| 10G2358F1 | ATTCTAGAAAGGCTATTCTCAAGTGTGGGG |
| 10G2358R1 | ATCTGCAGTCAGATCTTCACCTCCCGAACGTCA |
| 10GPC8RF1 | ATTCTAGAATGGATGCCGCAGCTCTTCAGTATGAGAACCAGAAGCTGGTGCAGCAATTG |
| 10GPC8RR1 | ATCTGCAGTCAGATCTTCACCTCCCGAACGTCACTCTGTCCAAAGGGGGTGCCGCTACCTGG |
