# Supplementary material for: Flowering-Related RING Protein 1 (FRRP1) Regulates Flowering Time and Yield Potential by Affecting Histone H2B Monoubiquitination in Rice (Oryza Sativa)
Source: PLoS One. 2016 Mar 2;11(3):e0150458. doi: 10.1371/journal.pone.0150458 (PMC4774988; doi:10.1371/journal.pone.0150458)
Supplement: S2 Table — (DOCX) [file pone.0150458.s002.docx]

**S2 Table. Values of several agronomic traits between transgenic lines and WT in rice**.

| Lines | grain width (cm) | 1000 grain weight (g) | Num of tillers per plant |
| --- | --- | --- | --- |
| WT | 0.35±0.01 | 22.39±1.16 | 13.13±1.65 |
| RL1 | 0.34±0.01 | 22.78±1.75 | 12.47±2.36 |
| RL2 | 0.34±0.02 | 23.39±1.42 | 11.13±2.87 |
| RL3 | 0.35±0.02 | 22.86±1.44 | 12.60±4.05 |

Values are means ± standard deviation (N =15).
